# Local anaesthesia training for undergraduate students – how big is the step from model to man?

**DOI:** 10.1186/s12909-018-1389-6

**Published:** 2018-12-14

**Authors:** Christian Knipfer, Maximilian Rohde, Nicolai Oetter, Tim Muench, Marco Rainer Kesting, Florian Stelzle

**Affiliations:** 1 Department of Oral and Maxillofacial Surgery, University Medical Center Hamburg Eppendorf, University of Hamburg, Martinistraße 52, 20246 Hamburg, Germany; 2 Department of Oral and Maxillofacial Surgery, University Medical Center Munich, Ludwig-Maximilian University Munich, Lindwurmstraße 2a, 80337 Munich, Germany; 3 Department of Oral and Maxillofacial Surgery, Erlangen University Hospital, Friedrich-Alexander University Erlangen-Nuremberg, Glückstrasse 11, 91054 Erlangen, Germany

**Keywords:** Dental education, Local anesthesia, Local injection, Training model, Practical training, Dental infiltration anesthesia, Block anesthesia

## Abstract

**Background:**

Local anesthesia is an important skill and a prerequisite for most dental treatments. However, the step from theory to application on the patient is huge for the novice. Hence, a mannequin training model course was developed and implemented into the existing local anesthesia curriculum in undergraduate dental students. It was the aim of this study to evaluate the relation between training-model and real-life anesthesia performance and to measure whether a gain in skill on the model translates to the actual patient situation.

**Methods:**

Thirty-six third-year students (14 males, 22 females, age 24 years±2.98) attended the four-day course comprising each 4 h of lectures and practical training. The student cohort gave subjective ratings about the didactical components of the course after attendance by using the TRIL questionnaire (TRIL-mod; University of Trier). At the end of the course the performance of each student in administering an inferior alveolar nerve (IAN) block on the training model as well as on a fellow dental student was investigated using a standardized checklist. To evaluate the successful performance, the in vivo IAN-block was assessed using subjective patient-feeling, the sharp-blunt test and an objective pain- and thermal sensitivity tester (PATH).

**Results:**

The course was rated with an average score of 5.25 ± 0.44 (range 1–6; 6 = best). On the training model, 69.4% of the students successfully performed an IAN-block. The in vivo assessment, objectified by the PATH test, showed a successful anesthesia in 36.9% of the cases. The assessment of local anesthesia by using the sharp blunt test and the subjective patient feeling significantly correlated with these findings (k = 0.453–0.751, *p* < 0.05). The model performance did not correlate with the performance on the patient (k = 0.137, *p* = 0.198).

**Conclusions:**

Although subjective ratings of the course were high, the anesthesia success rate on mannequin models did not imply an equal performance on the in vivo setting. As local anesthesia training models are a valuable didactic complement, the focus of the training should be on to the actual real life situation. Chair side feedback should be offered to the students using one of the presented evaluation methods.

## Background

Competence in the field of local anesthesia is regarded as a key skill of any dental practitioner. Successful pain management can not only facilitate the treatment for both the patient and the dental professional, but it has also been suggested, that patients may choose their dentist based on his or her ability to provide a pain-free therapy [1, 2]. With numbers quoting an annual usage of 300 Mio. cartridges of local anesthetic in the US and over 2 Million injections/day worldwide, it is one of the most common procedures in everyday dentistry [3, 4]. For interventions concerning the lower jaw, the conventional indirect or direct inferior alveolar nerve block will be the approach of choice for most practitioners [5].

As psychogenic reactions, such as anxiety-induced syncopes, hyperventilation, nausea, vomiting etc. are by far the most common adverse effects experienced in the administration of local anesthesia, the dentist should be competent and confident in the nerve block technique of his or her choice and project this confidence towards the patient [4, 6]. For the alveolar nerve block, however, a high failure rate ranging from 5 to 47% is reported in literature, especially when looking at sufficient anesthesia of the pulp [5, 7, 8]. Other techniques, such as the somewhat neglected Vazirani-Akinosi or Gow-Gates approaches, are sometimes quoted to be superior to the conventional techniques, but overall seem to permit a similar success rate [5, 9–12].

To face the challenges posed by the requirement of an adequate local anesthesia, the training of the theoretical principles and different techniques is a topic that is usually included in a dental student’s curriculum at a relatively early stage. Here, a wide range of teaching practices are reported, not always but preferably including an initial practical training on an anatomical reference model [13, 14]. In other fields of dentistry, e.g. tooth extractions, it has been shown that practical skill training on models can significantly improve the performance of the students prior to the transition to the real patient [15, 16]. In the case of local anesthesia no positive influence on the subjectively measured anesthesia success rate could be found, although training on a mock up model preceding the first actual injection was suggested to significantly boost the perceived confidence of the students, [17]. It is unclear, whether a gain in practical skills and success on the training model translates to the actual in vivo situation and how this in vivo success should best be measured in the context of a training course.

Therefore, after their course in theoretical and practical local-anesthesia training, the present study evaluated a student cohort’s performance on the training models and compared it to the objective real-life results using a standardized checklist. The in-vivo anesthesia-success (rate of successful anesthesia in fellow students) was measured using two subjective approaches (level of anesthesia reported by the treated students and sharp-blunt test) that were objectified with the Pain and Thermal Sensitivity nerve tester (PATH). Additionally, the student’s perception of the training course was assessed using a 5-topic questionnaire.

## Methods

### Test cohort

The test cohort comprised 36 third-year dental students (22 Female, 14 Male, 22-32a, Table 1) representing the entire class of the respective clinical semester.

**Table 1:** Overview of the test cohort, 3rd year dental students after mandatory anesthesia training course

Cohort	*n*	Age *(years)*			
		*mean*	*min*	*max*	*± SD*
all	36	24.33	22	32	2.986
male	14	24.93	22	32	3.075
female	22	23.95	22	31	2.935

*SD* Standard deviation

At this point of their studies they are at the beginning of the clinical and practical term and have not received any prior training in the field of local anesthesia.

### Anesthesia training course (mandatory

A newly developed local anesthesia training course was established 6 months before the investigations of this study. The course consists of 4 h of theoretical lectures and 12 h of practical training over a period of 4 days, the attendance is mandatory. At the end of the course, the students have to pass a written 20-question multiple choice exam to advance to the patient treatment part of their clinical term.

#### Lectures

The lectures are grouped in 4 daily blocks of 60 min each and given by a senior resident of the department of Oral and Maxillofacial Surgery (OMFS). The students are presented with the anatomical, medical, pharmacological, practical and legal aspects of the local anesthesia techniques relevant for dentistry. The slides of the lectures are made available online.

#### Practical training on mannequins

During the practical part of the training course, the different local anesthesia techniques are demonstrated on the mock-up training models by two senior residents of the OMFS department. The demonstrations include local infiltration anesthesia in the upper and lower jaw, block-anesthesia of the nasopalatinal and the palatinal nerve as well as the direct and indirect technique for the mandibular alveolar nerve block.

The training model used for this course consists of an upper head and body mannequin with a socket for the mounting of different jaw models (Frasaco, Tettnang, Germany). Successful injection is confirmed by a special anesthesia training model featuring simulated and anatomically correct nerve locations (AG 3 IB, Frasaco).

**Fig. 1:**
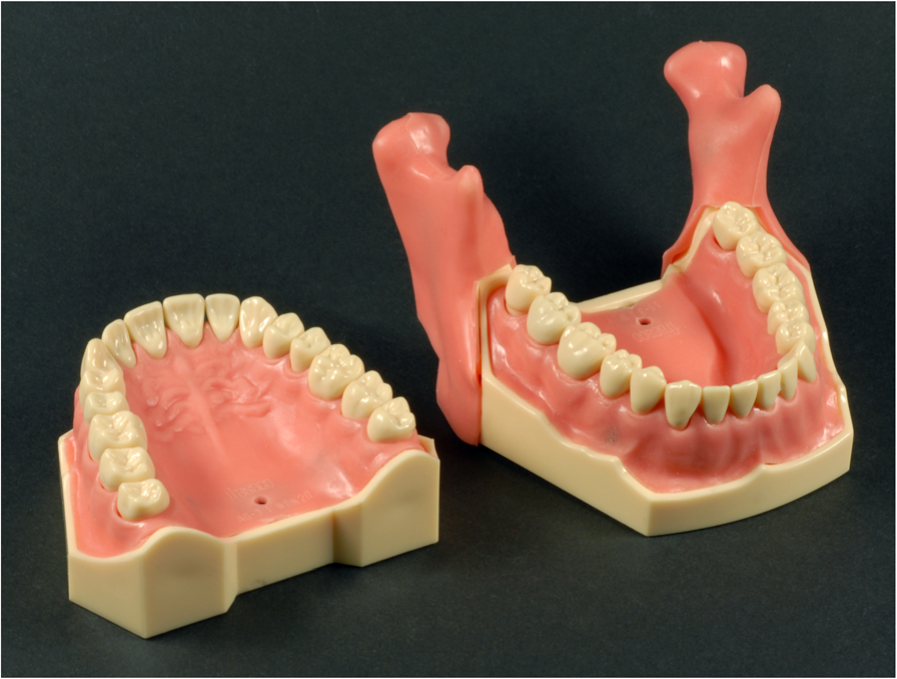
Anesthesia training jaw model

The battery-operated electronic typodont consists of an upper and lower jaw (articulating, stable intercuspidation). The jaws as well as the teeth (*n* = 32) are fabricated in hard thermosetting material. Gingivae are elastic and removable (replacement is possible according to wear). The integrated micro-electronic circuit has electric contact points in the maxilla (*n* = 3), the mandible (*n* = 2) and the ascending mandibular ramus (*n* = 2). The models give an acoustic signal when the correct region is perforated with a dry syringe (Fig. 1). The so equipped mannequins are strapped to a dental chair in order to simulate the patient’s and dentist’s position.

After demonstration of the different techniques, the students are asked to randomly form pairs of two and to perform and practice the previously demonstrated anesthesia methods on the models. Two senior OMFS residents and two tutoring 4th year students are available for further instructions, questions and support, giving a staff-to-student ratio of 1:9. In each pair, the student not performing the injection is asked to assist their fellow course attendee. This way, each attendee is obliged to perform the different techniques while simulating a real doctor-patient interaction.

#### Practical training on fellow students

During the last 2 h of each course day, as a mandatory part of the course, one of the senior resident demonstrates each infiltration anesthesia and direct and indirect alveolar nerve block techniques on the attendees. Here, the same procedure demonstrated with the help of the training models is followed.

The attendees subsequently are randomly assigned into groups of two and then asked to perform the same techniques on each other to train their skills on humans. Here, each step is supervised by one of the attending senior residents to offer assistance and answer questions. The course attendees are allowed to practice the techniques on the training models and fellow students each day during the practical course time.

### Course assessment questionnaire (voluntary)

For this study, at the end of the teaching course, the students were asked to anonymously rate the course using a modified TRIL-mod questionnaire, originally developed by the psychology department of the University Trier, Germany [18]. This modular and validated assessment tool originally comprises eight different chapters on (I) structure and didactics, (II) suggestion content, (III) communication skills, (IV) practical component, (V) motivation for course enrollment, (VI) overall rating of the course, (VII) an additional online tool and (VIII) room for open comments, where students can express criticism or make suggestions. Chapters five and seven of the original test were excluded in the present study, as they did not apply here: an online course was not offered and the participation was mandatory.

### Student skills assessment (voluntary)

#### Mannequin and mutual anesthesia

After successfully finishing the training course, the students were asked to take part in the practical evaluation of their acquired skills; all 36 agreed.

First, the students were asked to perform an inferior nerve block anesthesia on the familiar mannequin models using a technique of their choice and without any assistance from the evaluators. The injection success was assessed through the acoustic signal from the training model. The overall performance was rated with a standardized 7-topic checklist (Table 2). Here, all of the sub-items had to be performed correctly to score a point.

**Table 2:** Theoretical/practical skills checklist, all subitems have to be answered/performed correctly to gain 1 point

1.	*Ask patients medical history, for example:*
	• Prior allergic reactions
	• Cardiovascular conditions
	• Pregnancy
2.	• Correct preparation of the syringe (saline solution for Mock-Up Models)
	• Filling of the syringe
	• Draining of air before injection
	• Handling and positioning of the cannula
	• Answer one theoretical question about the local anesthetic (e.g. active agent concentration, vasoconstrictor concentration)
3.	• Correct position relative to the patient when administering the anesthetics
4.	• Correct execution of the chosen injection technique
	• Region of injection
	• Correctly describe the theoretical base of the chosen technique
5.	• Aspiration
	• Aspirate to prevent intravasal injection
6.	• General handling of instruments
	• Patient protection
	• Waste disposal
7.	• Infection prophylaxis
	• Wear personal safety equipment (gloves, glasses, facemask)
	• Correct use of sharps container
	• No recapping

The participants then were asked to perform an inferior nerve block on a fellow student (from here on called “patient”), again using a technique of their choice and without assistance from one of the evaluators. For this, 1.8 ml of an articaine compound containing a 1:100.000 adrenaline vasoconstrictor portion were administered (Ultracain DS, Sanofi-Aventis, Germany). Their performance was rated using the same 7-topic checklist, this time by another resident to avoid any bias during the re-evaluation of the same cohort. The success of the injection was evaluated using the tests described below.

#### Neurosensory testing of anesthesia performance

Pre-existing neurosensory disturbances were ruled out using the “sharp-blunt” test before the performance testing [19, 20]. All subjective and objective tests were carried out 20 min after the injection of the anesthetic and within a time-frame of 30 min according to the onset-time and effect-duration of the articaine compound [21].

The results were determined using the subjective rating of the “patients” giving quality and region of the anesthesia (subjectively sufficient/not sufficient and N. mandibularis/N.lingualis/both) and the same sharp-blunt test mentioned above. Here, the skin was gently pierced with either the sharp or blunt end of a conventional dental probe. The test was repeated for four times and the students were asked to describe the sensation as either “sharp” or “blunt”. The quality of the local anesthesia then was classified according to the correct answers: all correct = normal sensation, 3/4 = hypesthesia, 2/4 = paresthesia, 1/4 or 0/4 = anesthesia [22].

After this subjective rating, the students underwent a computer-based test of thermaesthesia, the “Pain and Thermal Sensitivity Test” (PATH, TSA 2011, Medoc Medical, Israel) to standardize and objectify the outcome of the local anesthesia with an independent method. The setup comprises a thermo-electrode (Peltier element) that allows for the administration of cold or warm stimuli ranging from 32 °C to 0 °C and 32 °C to 50 °C, respectively. When reaching the threshold for the perception of a change in temperature, the students are asked to press a button. This is, for each student, repeated for 10 times (5 cold, 5 warm) on the extraoral innervation region of the mandibular nerve on either side. The interval between stimuli is automatically varied by the computer system to avoid any subjective influence. Following Schultze-Mosgau et al., the decisive criterion for the degree of sensitivity loss was considered to be a side-to-side difference in the detection of the temperature change [22]. The threshold value for a significant anesthesia was set to _Δ_t = 15 °C, following the PATH systems computer output.

### Statistics

All statistical calculations were performed with SPSS version 21 (IBM, USA). Descriptive statistics, mean values, frequencies and the standard deviation were calculated where applicable. The transferability of the training-model results to the real patient was assessed using the McNemar test for connected dichotomous samples on the “Model: PATH” pair. The same test was applied for the comparison of the objective and subjective sensibility-tests, using the sample pairs “PATH: Subjective patient feeling” and “PATH: blunt-sharp-discrimination”. The agreement between the subjective and objective tests, as well as the model and patient performance were further evaluated with Cohens Kappa.

## Results

### Questionnaires

All of the 36 questionnaires were returned, the results are given in Table 3.

**Table 3:** Results of Checklist

Item No.	Item Topic	Mean Score ± SD (*N* = 36) best score = 6
1	Structure and Didactics	4.92 ± 0.59
2	Suggestion Content	5.08 ± 0.47
3	Communication Skills	5.27 ± 0.53
4	Practical Component	4.42 ± 0.42
5	Overall Rating	5.25 ± 0.44

*SD* Standard deviation

As can be seen from the results of the TRIL-questionnaire, the students overall rating of the training course was very good (5.25 ± 0.44 of 6). The “Practical Components” topic concerned with the practice-models and student-to-student anesthesia can be identified as the item with the lowest score (4.42 ± 0.42). This can be explained when looking at the comments given in the open section of the questionnaire: while 56% of the students stated a gain in confidence resulting from the mannequin training, 50% criticized the lack of anatomical landmarks and unrealistic depiction of the in-vivo situation, with 25% specifically naming the models very rigid mucosa as particularly irritating. Ten of the 36 students requested more training time on the models and a lower student to staff ratio.

### Mannequin and practical in-vivo anesthesia

The correct execution of the local anesthesia by the students on both, the mannequin models and their fellow students, was rated using the above-mentioned seven topic checklist. The results are given in Table 4.

**Table 4:** Assessment of Execution of local anesthesia with seven topic checklist

Topic			Mannequin Model *N* = 36		Fellow Student *N* = 36	
			*Correct*	*Incorrect*	*Correct*	*Incorrect*
*1*	*Medical history*		35 (97.2%)	1 (2.8%)	32 (88.9%)	4 (11.1%)
*2*	*Preparation of the syringe*		33 (91.7%)	3 (8.3%)	33 (91.7%)	3 (8.3%)
*3*	*Dentists’ position*		36 (100%)	0	36 (100%)	0
*4*	*Technique*	Direct	*N* = 19 (52.8%)		*N* = 30 (83,3%)	
			14 (73.3%)	5 (26.3%)	22 (73,3%)	8 (26,6%)
		Indirect	*N* = 17 (47.2%)		*N* = 6 (16.6%)	
			11 (64.7%)	6 (35.3%)	4 (66.7%)	2 (33.3%)
*5*	*Aspiration*		34 (94.4%)	2 (5.6%)	36 (100%)	0
*6*	*Handling of instruments*		35 (97.2%)	1 (2.8%)	34 (94.4%)	2 (5.6%)
*7*	*Infection protection*		32 (88.9%)	4 (11.1%)	34 (94.4%)	2 (5.6%)

Most students performed the tasks correctly. Medical history, syringe preparation, dentist’s position, aspiration, instrument handling and infection protection posed no major problems (max. Number incorrect = 4). The main issue in both, the ex-vivo as well as the in-vivo group showed to be the correct execution of the chosen anesthesia-technique.

On the training models, 19 (52.8%) students chose the direct technique while 17 (47.2%) students chose the indirect approach. Correct execution was found in only 25 cases (69.4%). The main problem named by the unsuccessful students was the insecurity where to place the needle for the direct technique and the very thick and rigid mucosa of the training model for the indirect technique. The theoretical questions were answered correctly by all of the participants.

Notably, after the transition to the in-vivo assessment, 30 out of 36 students (83.3%) preferred the direct approach over the indirect one. Correct execution of the techniques was found in 26 cases (72.2%). The main problem again was observed to be the insecurity concerning the correct positioning of the needle. When the students were asked why they chose the direct technique over the indirect one, most claimed the direct approach to be easier and more intuitive, while the indirect approach came with a subjective feeling of insecurity. As another factor, the difference between the depiction in the training models and the anatomical in-vivo situation was named.

The results concerning the injection success and the comparison of the different assessment techniques are given in Table 5. On the training model, 25 students (69.4%) achieved a successful anesthesia result indicated by the acoustic signal. This is in agreement with the observed number of correctly performed anesthesia techniques (*n* = 25, 69.4%).

**Table 5:** Injection success compared to different assessment techniques

Method	Correct Technique	Successful	Not Successful	McNemar Test: compared to PATH result (α = 5%) *p*=	Cohens Kappa Test: Compared to PATH result k=
Acoustic signal on Mannequin model	25 (69.4%)	25 (69.4%)	11 (30.6%)	0.004	0.198 *p* = 0.137
Objective: PATH-Result	26 (72.2%)	13 36.9%	23 63.1%		
Subjective: Affection of N. alveolaris or N. alveolaris and N. lingualis		19 (52.8%)	17 (47.2%)	0.109	0.453 *p* = 0.004
Subjective: Sharp-Blunt Differentiation		11 (30.6%)	25 (69.4%)	0.625	0.751 *p* ≤ 0.001

In the objective analysis of anesthesia performance, the students were not able to reproduce this result. Here, although 26 (72.2%) students performed their chosen technique correctly as rated by the evaluators, only 13 of the 36 participants (36.9%) were able to administer an objectively successful anesthesia, overall performing significantly worse than before (*p* = 0.004). Of the 25 students having success on the model, only 11 were able to repeat this on the “patient”. Although 19 “patients” (52.8%) stated to feel an affection of either the N. alveolaris inf. or both the N. alveolaris inf. and the N. lingualis, only 11 (30.6%) fulfilled the sharp-blunt test’s criteria for an anesthesia (0–1 correct out of 4). A McNemar test and Cohens kappa test showed that the results of the objective PATH test and both subjective assessments are in good agreement: the proportion of the two features were similar in the test pairs (p > > 0.05), k ranged from 0.453 to 0.751 (p < < 0.05). The subjective description would slightly overestimate the anesthesia effect (8 false positives, 2 false negatives), while the sharp-blunt test would underestimate it (3 false negatives, 1 false positive).

## Discussion

Local anesthesia training is an important topic in the dental student’s curriculum and entails both theoretical and practical knowledge in the field of peripheral nerve blocks. There are several different approaches to these teaching classes, ranging from only theoretical lessons to training on fellow students or training on special teaching models. For the transition from theoretical knowledge to practical execution, Brand et al. report that dentistry students usually perform their first injection in a human on a fellow dental undergraduate [13]. This practice has repeatedly been criticized as being unethical, and it has been shown that such training comes with great anxiety and stress for both the “recipient” and the “operator” [14, 23, 24]. As a consequence, dental schools and universities seek to include model-training into their local anesthesia courses to allow for further training prior the first in-vivo injection [13]. At the same time, a high percentage of the students themselves wish for the introduction of anesthesia training models to practice their skills before setting their hands on a real human [25].

The results found in this study however suggest that, even after thorough mannequin training, this transition from model to patient still proves to be difficult. Although the course was rated well by the students and most of the tasks coming with the administration of a local anesthetic are mastered (Tables 3 and 4), the anesthesia technique itself seems to be more challenging. The success on the training model often was not transferrable to the patient situation and allowed for no conclusions concerning the expected in-vivo performance (k = 0.198, *p* = 0.137).

Within this study the success rate is a proper indicator for knowledge-transfer and growth and allows conclusions on the anesthesiological performances within the group of dental students. However, a comparison to professional dentists’ performances in real patients and to results of other studies investigating success rates of dentists is not sensible or possible. This drawback may result from the high number of influencing factors on the strength and mode of action of local anesthetics [26–29]. It also has to be noted that the results were gathered from a relatively small group of only 36 students and that the validity of the observations should be verified by further studies using lager collectives. Additionally, the effectiveness of the anesthesia was measured using the PATH test described above. This method is mainly meant for peripheral nerve function assessment, in this case of the inferior mandibular nerve. A pulp tester, thermal or electrical, might have better simulated the later situation in the dental practice, where the main goal usually is painlessness of the teeth achieved by pulp anesthesia. In order to keep potential influencing factors such as dental fillings to a minimum, it was decided to pursue the approach using direct pain and thermal sensitivity testing of the IAN. The outcome of the present study is however confirmed by Brand et al., who found that mannequin-training or no mannequin training does *not* make a significant difference in the anesthesia performance on the patient [17].

In both studies, the students explicitly criticized the models inaccurate depiction of the human anatomy, especially the lack of the pterygomandibular plica. This may cause problems in the transfer of theoretical knowledge from lectures to practical skills on the model and again from the skills gained on the model to the patient situation. In the same context, Yetka et al. found that a more accurately sculpted and anatomically realistic training model is considered more helpful by the students and at the same time may increase the injection success [30].

However, even though the number of correct injections performed by the students in this cohort are equally high in the model and the patient, this does not reflect in the objective or subjective anesthesia success. This high discrepancy may be explained by the difficulty to entirely rate, observe and evaluate the students’ injection attempt without causing any bias by offering support: the evaluators were only able to rate the injection site and the theoretical knowledge of the students. Other, non-observable factors (e.g. penetration depth) may influence the anesthesia success.

It should be noted that the training models, as described above, can only provide basic feedback about the correct injection site and penetration depth for the trainees and evaluators. The acoustic signal can help the students find the right position of the syringe during initial attempts. Due to the technical specifications of the training model there is no possibility to mute the models for the trainees while allowing the evaluators to observe the success of the attempt. This implies that the students may simply advance the needle until they hear the sound indicating a “successful” injection (viz. correct injection site and penetration depth), without this having a verifiable teaching effect. If this was the case it could be an additional barrier for the translation from model to man in this study and also explain the relatively higher success rate on the model.

Additionally, as summarized by Palti et al., common sources of error include the lack of knowledge or variations in the anatomy, technical errors, inflammation and infection, inadequate mouth opening, a wrong positioning of the needle, needle deviation and, especially relevant here, the lack of experience and anxious patients [2, 31–33]. It has to be kept in mind that the here evaluated procedure was the first truly unassisted local nerve-block anesthesia for all participants and that thus anxiety and insecurity will be a factor. Concerning the evaluation of the students’ practical skill on the patient, the two subjective approaches, patient-feeling and sharp-blunt-discrimination showed to be equally feasible (k = 0.453–0.751, *p* < 0.05), with the sharp-blunt-discrimination more accurately reflecting the results of the objective PATH-test. As the training model performance does *not* seem to be a probate instrument to predict and measure the in-vivo anesthesia performance, maybe feedback using one of the three above-mentioned sensibility-test should be offered to the students. This may prevent the development of further insecurities, when the basically well-mastered technique on the model does not produce the anticipated results on the patient. The results also suggest that the anesthesia training should not be considered completed with the end of the training course, but that supervised teaching and coaching must be continued by the then responsible department (e.g. conservative dentistry, prosthetics) during the transition to patient treatment in the early clinical terms.

## Conclusions

The combination of theoretical lessons, practical mannequin training and practical demonstrations seems to be a promising teaching method looking at the general tasks associated with performing a local block anesthesia, such as observing hygiene requirements or obtaining the patients’ medical history. In particular, the exact anamnesis with regard to allergies and physical characteristics (pregnancy, hypertension etc.) can be of major significance for the imminent local anesthesia.

The success rate of the IAN block itself however shows significantly different results when comparing model-performance to in-vivo performance. The rate of successful inferior nerve-blocks on the mannequin-training models is much higher and unfit to reliably predict the same students’ performance on the patient. One major problem commented on by the students proved to be the models’ inaccurate reconstruction of the human anatomy. This suggests that, when training models are used in an anesthesia training course, they should accurately reflect the physique of the oral cavity to prevent difficulties in the transfer from model to patient.

For measuring the in-vivo anesthesia success, subjective patient-feeling, sharp-blunt-discrimination and objective PATH-testing proved to be equally reliable, with the sharp-blunt test probably offering the best tradeoff between accuracy and simplicity. This feedback should ideally already be offered to the students during the training courses. Anesthesia teaching and coaching must continue during the clinical terms and on the patient, as mannequin-training does not seem to adequately prepare the students for the real-live situation.

## Abbreviations

a: Years (age)
IAN: Inferior alveolar nerve
OMFS: Oral and maxillofacial surgery
PATH: Pain- and thermal sensitivity tester

## References

[1] Johnson TM, Badovinac R, Shaefer J (2007). Teaching alternatives to the standard inferior alveolar nerve block in dental education: outcomes in clinical practice. J Dent Educ.

[2] Palti DG, Almeida CM, Rodrigues Ade C, Andreo JC, Lima JE (2011). Anesthetic technique for inferior alveolar nerve block: a new approach. J Appl Oral Sci.

[3] Milgrom P, Fiset L (1986). Local anaesthetic adverse effects and other emergency problems in general dental practice. Int Dent J.

[4] Haas DA (2002). An update on local anesthetics in dentistry. J Can Dent Assoc.

[5] Goldberg S, Reader A, Drum M, Nusstein J, Beck M (2008). Comparison of the anesthetic efficacy of the conventional inferior alveolar, Gow-gates, and Vazirani-Akinosi techniques. J Endod.

[6] Plasschaert AJ, Holbrook WP, Delap E, Martinez C, Walmsley AD (2005). Profile and competences for the European dentist. European journal of dental education : official journal of the Association for Dental Education in Europe..

[7] Kaufman E, Weinstein P, Milgrom P (1984). Difficulties in achieving local anesthesia. J Am Dent Assoc.

[8] Wong MK, Jacobsen PL (1992). Reasons for local anesthesia failures. J Am Dent Assoc..

[9] Gow-Gates GA (1973). Mandibular conduction anesthesia: a new technique using extraoral landmarks. Oral Surgery, Oral Medicine, Oral Pathology.

[10] Cruz E, Quengua J, Gutierrez I, Abreu M, Uy H (1993). A comparative study: classical, Akinosi, and Gow-gates techniques of mandibular nerve block. The Journal of the Philippine Dental Association.

[11] Sisk AL (1986). Evaluation of the Akinosi mandibular block technique in oral surgery. J Oral Maxillofac Surg.

[12] Todorović L, Stajčić Z, Petrović V (1986). Mandibular versus inferior dental anaesthesia: clinical assessment of 3 different techniques. Int J Oral Maxillofac Surg.

[13] Brand HS, Kuin D, Baart JA (2008). A survey of local anaesthesia education in European dental schools. European journal of dental education : official journal of the Association for Dental Education in Europe.

[14] Chandrasekaran B, Cugati N, Kumaresan R (2014). Dental Students' perception and anxiety levels during their first local anesthetic injection. Malays J Med Sci.

[15] Marras I, Nikolaidis N, Mikrogeorgis G, Lyroudia K, Pitas I (2008). A virtual system for cavity preparation in endodontics. J Dent Educ.

[16] Stelzle F, Farhoumand D, Neukam FW, Nkenke E (2011). Implementation and validation of an extraction course using mannequin models for undergraduate dental students. Acta Odontol Scand.

[17] Brand HS, Baart JA, Maas NE, Bachet I (2010). Effect of a training model in local anesthesia teaching. J Dent Educ.

[18] Gollwitzer M, Schlotz W, Krampen G, Zayer H (2003). Das “Trierer Inventar zur Lehrveranstaltungsevaluation” (TRIL): Entwicklung und erste testtheoretische Erprobungen. Psychologiedidaktik und Evaluation IV.

[19] Takeuchi T, Furusawa K, Hirose I (1994). Mechanism of transient mental nerve paraesthesia in sagittal split mandibular ramus osteotomy. Br J Oral Maxillofac Surg.

[20] Chen N, Neal CE, Lingenbrink P, Bloomquist D, Kiyak HA (1999). Neurosensory changes following orthognathic surgery. Int J Adult Orthodon Orthognath Surg.

[21] Kanaa MD, Whitworth JM, Meechan JG (2012). A comparison of the efficacy of 4% articaine with 1:100,000 epinephrine and 2% lidocaine with 1:80,000 epinephrine in achieving pulpal anesthesia in maxillary teeth with irreversible pulpitis. J Endod..

[22] Schultze-Mosgau S, Krems H, Ott R, Neukam FW (2001). A prospective electromyographic and computer-aided thermal sensitivity assessment of nerve lesions after sagittal split osteotomy and Le fort I osteotomy. J Oral Maxillofac Surg.

[23] Rosenberg M, Orr DL, Starley ED, Jensen DR (2009). Student-to-student local anesthesia injections in dental education: moral, ethical, and legal issues. J Dent Educ.

[24] Hossaini M (2011). Teaching local anesthesia in dental schools: opinions about the student-to-student administration model. J Dent Educ.

[25] Brand HS, Tan LL, van der Spek SJ, Baart JA (2011). European dental students’ opinions on their local anaesthesia education. European journal of dental education : official journal of the Association for Dental Education in Europe.

[26] Sawadogo A, Coulibaly M, Quilodran C, Bationo R, Konsem T, Ella B. Success rate of first attempt 4% articaine para-apical anesthesia for the extraction of mandibular wisdom teeth. Journal of stomatology, oral and maxillofacial surgery. 2018. 10.1016/j.jormas.2018.06.005 29936238

[27] Haghighat A, Jafari Z, Hasheminia D, Samandari MH, Safarian V, Davoudi A (2015). Comparison of success rate and onset time of two different anesthesia techniques. Medicina oral, patologia oral y cirugia bucal.

[28] Saatchi M, Shafiee M, Khademi A, Memarzadeh B (2018). Anesthetic efficacy of Gow-gates nerve block, inferior alveolar nerve block, and their combination in mandibular molars with symptomatic irreversible pulpitis: a prospective. Randomized Clinical Trial J Endod.

[29] St George G, Morgan A, Meechan J, Moles DR, Needleman I, Ng YL (2018). Injectable local anaesthetic agents for dental anaesthesia. The Cochrane database of systematic reviews.

[30] Yekta SS, Lampert F, Kazemi S, Kazemi R, Brand HS, Baart JA (2013). Evaluation of new injection and cavity preparation model in local anesthesia teaching. J Dent Educ.

[31] DeSantis JL, Liebow C (1996). Four common mandibular nerve anomalies that lead to local anesthesia failures. J Am Dent Assoc..

[32] Wilson S, Johns P, Fuller PM (1984). The inferior alveolar and mylohyoid nerves: an anatomic study and relationship to local anesthesia of the anterior mandibular teeth. J Am Dent Assoc..

[33] Steinkruger G, Nusstein J, Reader A, Beck M, Weaver J (2006). The significance of needle bevel orientation in achieving a successful inferior alveolar nerve block. J Am Dent Assoc..

